# The Role and Involvement of Stem Cells in Periodontology

**DOI:** 10.3390/biomedicines11020387

**Published:** 2023-01-28

**Authors:** Ancuta Goriuc, Liliana Foia, Karina Cojocaru, Diana Diaconu-Popa, Darius Sandu, Ionut Luchian

**Affiliations:** 1 Department of Biochemistry, Faculty of Dental Medicine, “Grigore T. Popa” University of Medicine and Pharmacy, 16 Universității Street, 700115 Iași, Romania; 2 Department of Dental Technology, Faculty of Dental Medicine, “Grigore T. Popa” University of Medicine and Pharmacy, 16 Universității Street, 700115 Iași, Romania; 3 Department of Periodontology, Faculty of Dental Medicine, “Grigore T. Popa” University of Medicine and Pharmacy, 16 Universității Street, 700115 Iași, Romania

**Keywords:** periodontitis, stem cells, periodontal regeneration, dental mesenchymal stem cells, non-dental stem cells, induced pluripotent stem cells

## Abstract

Periodontitis is a widespread inflammatory condition, characterized by a progressive deterioration of the supporting structures of the teeth. Due to the complexity of periodontal tissue and the surrounding inflammatory microenvironment, the repair of lesions at this level represents a continuous challenge. The regeneration of periodontal tissues is considered a promising strategy. Stem cells have remarkable properties, such as immunomodulatory potential, proliferation, migration, and multilineage differentiation. Thus, they can be used to repair tissue damage and reduce inflammation, potentially leading to periodontal regeneration. Among the stem cells used for periodontal regeneration, we studied dental mesenchymal stem cells (DMSCs), non-dental stem cells, and induced pluripotent stem cells (IPSCs). Although these cells have well documented important physiological characteristics, their use in contemporary practice to repair the affected periodontium is still a challenge.

## 1. Introduction

Periodontitis is an infectious and inflammatory condition with an impact on the immune system and can lead to the destruction of periodontal tissue and eventually to the loss of teeth [1]. Practicing dentists can treat this condition with conventional therapies; however, restoration of the damaged periodontium is not yet feasible. Regenerative periodontal therapy has attempted to rebuild periodontal supporting tissues, including alveolar bone, gingiva, periodontal ligaments (PDLs), and cementum. In addition to tissue regeneration, damaged periodontal tissues can be repaired by applying stem cells, growth factors, or an extracellular matrix scaffold [2]. Among the stem cells studied thus far, those that have proven to have an important role in the regeneration of periodontal tissues are mesenchymal stem cells (MSCs), embryonic stem cells (ESCs), and induced pluripotent stem cells (iPSCs). MSCs are preferred for use in regeneration because they do not present ethical problems in the manner of ESCs [3]. In addition, they have been shown to contribute to periodontal regeneration when transplanted into periodontal bone defects, being isolated from bone marrow mesenchymal stem cells (BMSC). However, this technique has not proven to be advantageous because of the associated pain during harvesting and the low number of harvested cells; therefore, researchers have tried to collect stem cells from dental tissues, such as the ligament, the gum, dental follicles, dental pulp, apical papilla, and exfoliated human deciduous teeth [4,5].

Contemporary investigations based on stem cells refer to tissue engineering methods aiming to regenerate periodontal tissues, which include the regenerative processes that can occur at the level of the periodontium and the entire tooth (dental pulp, root, dentine, and alveolar bone) [4,6,7].

## 2. Pathological Processes Involved in Periodontitis

**Figure 1:**
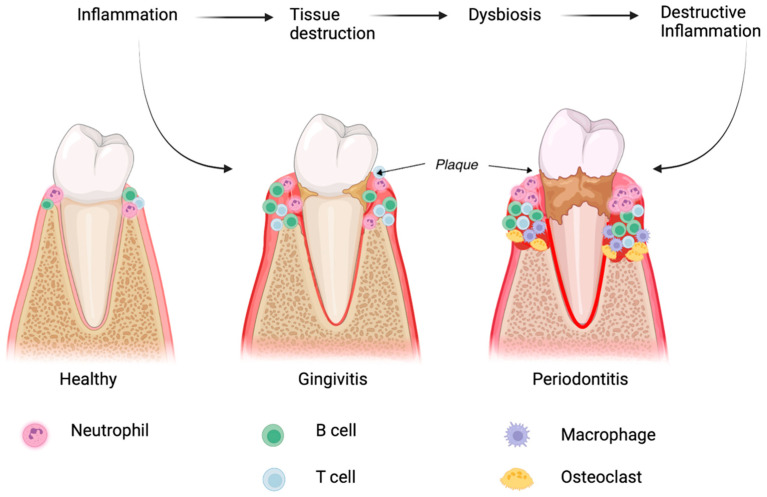
The role of dysbiosis in the development of periodontitis. The figure shows the influence of bacterial plaque in the development of periodontal disease (the brown spot around the tooth neck) as well as the immune response of the host (the types of cells that develop at the periodontal level).

The homeostasis of periodontal tissue depends on the fragile equilibrium between the immune system of the host and microbial attacks. Understanding the main patterns of inflammatory responses and their mechanisms at this level will facilitate the use of stem cells in the future treatment of this pathology [8,9]. Due to microbial imbalances and the susceptibility of the host, an inflammatory response can occur which involves the host’s immune system [10]. This response determines subsequent alterations of the support structures of odontal units, resulting in the onset of periodontal disease (Figure 1).

### 2.1. Microbial Dysbiosis and Its Role in Periodontitis

Among the microorganisms that can contribute to microbial dysbiosis at periodontal level, the most predominant are the anaerobic ones, such as Proteobacteria, Firmicutes, Spirochaetes, Synergistetes, and Bacteroidetes [11]. The presence of inflammatory mediators in large quantities at the subgingival level provides a specific microenvironment for the development of microbial colonies. Dysbiotic microbial colonies undermine immune responses of the host by initiating synergistic interactions that can trigger proteomic responses in order to acquire the necessary nutrients [11,12]. The dysbiotic oral microbiota derive their nutrients from the destroyed inflamed tissues, including degraded collagen peptides and hemoproteins [13]. Periodontopathogenic bacteria can improve their living conditions by modifying the host’s immune response. For example, these bacteria are not affected by neutrophil-mediated attack, and can thus protect themselves from complement systems. Therefore, neutrophils become unable to oppose the attack of these microorganisms that produce dysbiosis, the consequence being periodontal tissue degradation (Figure 1) [14].

The initiation of the inflammatory process and the progressive destruction of periodontal tissues also depend on the host’s susceptibility to periodontal damage [15,16]. The evolution grade and the destruction stage of periodontitis are based both on host factors, including the dysregulation of immunoregulatory mechanisms, immunodeficiencies, systemic diseases affecting the periodontal tissues (such as diabetes, arthritis, depression, cardiovascular disease, osteoporosis, obesity, or Alzheimer’s disease), and risk factors that can affect the host’s immune response, such as smoking, stress, aging, and microbial factors [17,18]. Defective immune responses of the host will determine their inability to inhibit the microbial colonies that cause dysbiosis; thus, a pathogenic cycle is initiated which is self-accentuating [10].

### 2.2. The Immune Response in Periodontal Diseases

Once the dysbiotic microbiota triggers the onset of periodontitis, the immune response turns from acute to chronic, causing periodontal degradation [19]. Periodontal pathogenic microorganisms can increase their resistance to the host’s immune attacks by interacting with neutrophil cells and the complement system [9]. In the gingival sulcus, neutrophils are recruited together with T cells, B cells, and monocytes.

Neutrophils are responsible for degrading protein from the bacteria membrane. They achieve this through the synthesis of elastase, an enzyme that causes the destruction of elastin and type IV collagen at the level of periodontium, ultimately leading to periodontal pockets [20]. Moreover, neutrophils synthesize cytotoxic substances and hydrolytic enzymes such as matrix metalloproteinases (MMPs) or reactive oxygen species (ROS) that are frequently involved in the destruction of periodontal tissues and in the occurrence of inflammation at this level. Cytotoxic substances and hydrolytic enzymes such as MMPs and ROS secreted by neutrophils frequently determine the destruction and inflammation of the adjacent inflammatory tissues [21]. They also release an activator of the nuclear factor kappa-B ligand receptor (RANKL) involved in osteoclastogenesis and periodontal bone resorption [22]. RANKL is recognized as a type II membrane protein and is classified as a member of the tumor necrosis factor (TNF) superfamily. RANKL is known to affect the immune system and regulate bone regenerative processes as well as bone re-modeling. RANKL also contributes to the regulation of apoptosis and osteoprotegerin (OPG), thus controlling cell proliferation.

B and T cells from inflammatory lesions are a major source of RANKL [23]. Neutrophils specifically mediate the chemotactic attraction of T helper (Th) 17 cells which, in turn, are mediated by interleukin-17 (IL-17). Th17 cells with a role in osteoclastogenesis secrete chemokines such as Chemokine receptor (CCR) 2, CCR 6, and CCR 20 [24,25]. The inflammatory infiltrate that determines the progression of the inflammatory process in the periodontal tissues mainly contains B cells, although plasma cells and a large number of immune complexes, as well as fragments of the complement system, are also present [26]. The secretion of MMPs and inflammatory cytokines by B cells induces the destruction of periodontal tissues [27].

Macrophages also play a role in remodeling connective tissue through maintaining a balance between MMPs and their tissue inhibitors (TIMPs). They also balance the activity of osteoblasts and osteoclasts, thus regulating homeostasis at the bone tissue level. Moreover, this ability of polymorphonuclear leukocytes and monocytes is determined by the secretion of various cytokines, such as tumor necrosis factor α (TNF-α), adhesion molecules, IL-1β, and IL-6. These factors determine the adhesion of cells to the endothelium, leading to an increase in the permeability of gingival capillaries and alveolar bone resorption [8,28].

## 3. Characteristics, Immunological Features, and the Evaluation of Periodontal Stem Cell Regeneration

This section depicts the characteristics and properties of stem cells: Dental Mesenchymal Stem Cells (DMSCs), non-dental stem cells, and iPSCs. In particular, the regenerative potential of each cell type for the affected tissues will be presented (Table 1).

### 3.1. Dental Mesenchymal Stem Cells (DMSCs)

DMSCs are classified into dental follicle stem cells (DFSCs), dental-pulp-derived stem cells (DPSCs), stem cells from apical papilla (SCAPs), gingival mesenchymal stem cells (GMSCs), stem cells from exfoliated deciduous teeth (SHEDs), dental socket stem cells (DSSCs), and ligament stem cells (PDLSCs) [1,29]. DMSCs are pluripotent stem cells with a potential for renewal and differentiation on multiple cell lines. DMSCs modulate the activity of various cell types involved in immunity. The immunological potential of DMSCs is based on numerous inflammatory factors secreted by cells with a role in immunity [7].

#### 3.1.1. Dental Follicle Stem Cells (DFSCs)

DFSCs are cells present in the dental follicle of the dental germ and are derived from the neural crest. If they are in a specific environment, DFSCs can differentiate into bone cells, adipocytes, chondrocytes, neurons, and cardiomyocytes. Recently, it was discovered that these cells have the ability to differentiate into salivary gland cells and ductal cells [53]. DFSCs are known for the formation of the periodontium by means of migration around the tooth bud and their differentiation into PDLs, osteoblasts, and cementoblasts. The surface cluster of differentiation (CD) markers of DFSCs are CD13, CD44, CD73, CD105, CD56, CD271, human leukocyte antigen (HLA-ABC), STRO-1, and Neurogenic locus notch homolog protein 1 (NOTCH-1). Of these markers, STRO-1 and CD44 can be used to identify DFSCs) [31].

The immunological suppressive properties of DFSCs are correlated with Toll-like receptors (TLRs). Fusobacterium nucleatum and Porphyromonas gingivalis can stimulate the expression of TLR2/TLR4 in the membrane of DFSCs, determining the inhibition of the proliferation of PBMCs [54]. In addition, DFSCs can stimulate the secretion of the anti-inflammatory cytokine IL-10 and simultaneously inhibit the pro-inflammatory markers affecting bacterial adhesion (IL-4, IL-8, and IFN-γ). DFSCs suppress bone degradation by modulating the phagocytic and chemotaxis activity and inducing macrophage polarization towards the M2 phenotype [55].

Several experiments on laboratory animals have proven that DFSCs have the ability to restore periodontal defects. For example, in an animal model with periodontal disease, DFSCs were implanted in the affected area, leading to new PDLs, cementoblasts, and osteoblasts. Other recent work has shown that the ectopic transplantation of DFSCs belonging to the human third molar, transplanted into mice, created a cementum–PDL complex [29,56]. Further, Ma et al. recently showed that the implantation of DFSCs-sEVs (cell-derived small extracellular vesicles) type cells in a periodontal defect improved the migration, proliferation and osteogenic differentiation of PDLSCs. Therefore, the use of DFSCs- sEVs for periodontal regeneration may have significant potential [57].

It is been known that the optimal use of sEVs for periodontal regeneration depends on the source of stem cells and their culture conditions. Therefore, this new therapeutical approach has obvious benefits, because these cells are easy to handle and reduce the risk of tumorogenesis, rejection by the host and the occurrence of subsequent infections [58]. Previous studies have shown that sEVs used in periodontal regeneration acted by inhibiting the expression of inflammatory cytokines and improving the osteogenic capabilities of bone marrow-derived mesenchymal stem cells (BMMSCs) [59]. Likewise, sEVs derived from dental stem cells (DSC) can be used to improve bone defects at the periodontal level through a newly discovered mechanism based on the mediation of cellular communication between osteoclasts and osteoblasts. Osteoblasts release RANKL-containing sEVs that will be transferred to osteoclast precursor cells. Afterwards, they transform into osteoclasts and stimulate the Osteoprotegerin/Receptor activator of the NF-κB ligand/receptor activator of the NF-κB (OPG/RANKL/RANK) signaling pathway, which is extremely important in bone metabolism [60].

#### 3.1.2. Dental Pulp Stem Cells (DPSCs)

Dental pulp stem cells are a population of stem cells derived from dental pulp extracted from human permanent teeth. These cells demonstrate phenotypic and functional properties very similar to those of bone-marrow-derived mesenchymal stem cells. DPSCs have the ability to transform into odontoblasts, osteoblasts, chondrocytes, adipocytes, or neurogenic cells, and exhibit a high expression of MSC surface markers [61]. DPSCs have the potential to mediate both innate and the specific immune responses through their interactions with T cells, B lymphocytes, macrophages, and NK cells. DPSCs exert immunological effects by inhibiting the proliferation of activated T cells and triggering cell apoptosis [34]. DPSCs suppress the production of immunoglobulins by B lymphocytes and the production of IL-17, while stimulating the secretion of IFNγ. DPSCs have an inhibitory effect on the proliferation of activated PBMCs, obtained through the generation of TGF [62]. DPSCs exert an anti-inflammatory function by means of two mechanisms. IDPSCs inhibit TNF-α secretion of macrophages through an IDO-dependent pathway, and also initiate the polarization of M2 macrophages. The induction of DPSC differentiation potentiates their inhibitory effect on NK-mediated cell lysis and cytotoxicity [63].

Park et al. showed that DPSCs repaired periodontal defects to a small extent, having a limited ability to form cementum-like structures compared with PDLSCs, which determined periodontal regeneration with bone, cementum, and Sharpey’s fibers. Few studies have characterized the role of DPSCs on pulp repair and highlighted the immunomodulatory properties in PDL-type tissues. Current evidence suggests that DPSCs are not an option in periodontal tissue engineering [29]. Although DPSCs have an important role in cementum or PDL regeneration, unknown factors are significantly involved. Recent studies have shown that PDLSCs provide a superior periodontal regeneration capacity compared to DPSCs. Future studies should focus on the types of growth factors or matrices which can improve their periodontal regeneration capacity [64].

#### 3.1.3. Stem Cells from the Apical Papilla (SCAPs)

**Figure 2:**
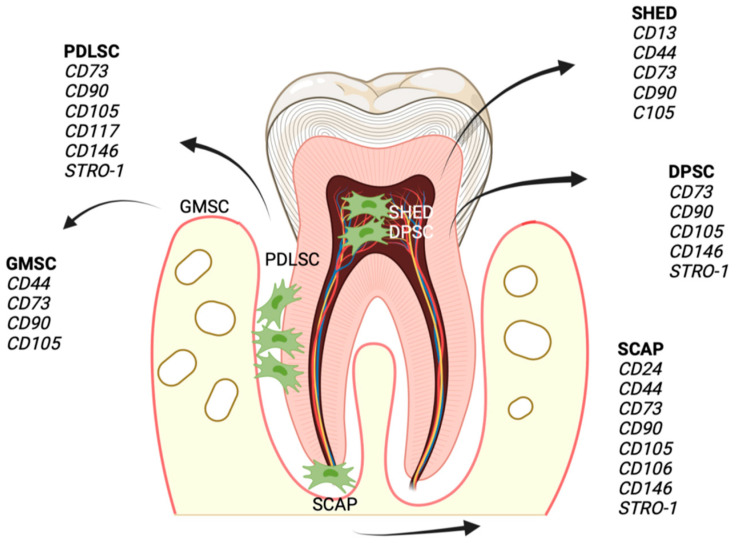
The distribution of DMSCs, their original place and surface markers.

SCAPs were initially isolated from the apical papillary tissue of immature teeth in 2006 (65). SCAPs are characterized by an increased self-renewal capacity, high proliferation potential, low immunogenicity, and multilineage differentiation ability. STRO-1, CD24, CD166, CD73, CD90, and CD146 are identified as surface markers of SCAPs (Figure 2). Among the main actions of SCAPs is the inhibition of T lymphocyte proliferation through an apoptosis-independent mechanism [37].

Moreover, the transplantation of SCAPs in an area with periodontal damage significantly improves certain parameters characteristic of the pathology 12 weeks after transplantation. Based on these characteristics, it has been suggested that SCAPs represent a promising source for periodontal repair [65].

#### 3.1.4. Gingiva-Derived Mesenchymal Stem Cells (GMSCs)

Epithelial and connective tissues comprise the human gingiva, which represents a main constitutive element of the periodontium; it exerts remarkable functions on periodontal regeneration and stands out for its scar-free wound healing properties. 

GMSCs were identified from gingival tissue (lamina propria) in 2009 [5]. After ascertaining their remarkable self-renewal, regeneration, and multilineage differentiation abilities, GMSCs now represent an adequate cell source in the treatment and engineering of periodontal tissues. In recent years, the immunomodulatory properties and accessibility of GMSCs have generated increased interest in the use of cell therapy [66]. Interactions between GMSCs and the inflammatory environment are achieved through the expression of TLRs 1, 2, 3, 4, 5, 6, 7, and 10, which influence the immunological properties of GMSCs. Human GMSCs can inhibit the activity of M1 macrophages through the production of Prostaglandin E2 (PGE2), IL-10, or IL-6. In addition, GMSCs significantly reduce DC activation and maturation by means of a PGE2-associated mechanism which suppresses the antigen-presenting capacity of DCs and significantly decreases the inflammatory response. Human GMSCs have inhibitory abilities on T cell activation by upregulating the immunosuppressive factors (IDO and IL-10) [39]. Several studies support the use of GMSCs in periodontal pathology. In an animal study, the grafting procedure of GFP-labelled GMSCs at the level of furcation lesions markedly improved the regeneration of periodontal tissues [67].

#### 3.1.5. Human Exfoliated Deciduous Teeth Stem Cells (SHEDs)

Dental pulp is a highly vascularized connective tissue, responsible for tooth homeostasis, and is sensitive to external stimuli. SHEDs were initially described and isolated from the exfoliated deciduous teeth. SHEDs have the potential to regenerate bone and tissues such as dentin, with a high osteoinduction potential and an increased proliferation rate [62].

SHEDs demonstrate immunomodulatory capacities by mediating the activation, maturation, and differentiation of T lymphocytes. They have an inhibitory effect on Th17 lymphocytes and a stimulatory effect on regulatory T lymphocytes [68]. In addition, SHEDs can inhibit the secretion of inflammatory markers (IL-2, TNF-α, and IFN-γ) and can stimulate the generation of IL-10, an anti-inflammatory factor [69]. SHEDs induce the polarization of bone marrow macrophages towards M2, generating anti-inflammatory effects on the periodontal tissue and regeneration of the periodontium. In an experimental model of periodontitis, the presence of SHEDs in the periodontal tissue led to a decrease in cytokine expression, gingival bleeding, and the establishment of new connections between the PDL and the alveolar bone. In conclusion, SHEDs contribute to the recovery of periodontal regeneration and decrease tissue inflammation at this level [70].

#### 3.1.6. Periodontal Ligament Stem Cells (PDLSCs) 

PDL is a type of connective tissue that ensures the connection between the tooth root and the neighbouring alveolar bone. The PDL originates in the dental follicle, having an important role in maintaining dental homeostasis and ensuring nutritional intake [3]. Initially, PDLSCs were identified in adults in the third molar. PDLCs have the ability to generate PDLs, cementum, alveolar bone, blood vessels and peripheral nerves [49]. These cells have a high capacity for renewal and proliferation. PDLSCs express many types of markers (CD13, CD 44, CD73, CD90, CD105) (41). Additionally, these cells do not express a series of hematopoietic markers such as CD14, CD19, CD40, CD45, CD 80 and CD86, but instead express antigens such as TRA-1-60, TRA1-81, sex determining region Y- box (Sox) 2, and alkaline phosphatase (ALP) [50,51].

Recently, PDLSCs have been proposed as promising cell sources for the repair of certain bony defects caused by periodontitis, due to their immunomodulatory properties. PBMCs generate IFN-γ and cause PDLSCs to secrete soluble factors such as TGF-β, indoleamine and hepatocyte growth factor, which reduce the proliferation of PBMCs. Neutrophil proliferation and apoptosis constitute another mechanism mediated by PDLSCs. PDLSCs inhibit the proliferation of T lymphocytes by reducing the secretion of glycoprotein 1b of the major histocompatibility complex and PGE2 originating from dendritic cells [43]. Additionally, PDLSCs improve the proliferation of anti-inflammatory Treg cells and inhibit pro-inflammatory Th1/Th2/Th17 lymphocytes [71]. The immunosuppression mechanism is mediated by PDLSCs by inhibiting the proliferation, migration and differentiation of B lymphocytes. PDLCSs can potentiate the anti-inflammatory phenotype by stimulating arginase 1, CD163, IL-10 and inhibiting TNF-α [4].

Numerous experiments carried out on laboratory animals, as well as clinical trials, have showed that PDLSCs can successfully induce the periodontal regeneration of certain defects. For example, applying PDLCs at the level of a periodontal defect in mice improves periodontal regeneration through the formation of PDLs, cementum-like tissue and new bone in the absence of inflammation [72]. PDLSCs have the ability to regenerate periodontal tissue in the absence of adverse effects. When porcine laboratory animal models with periodontitis received allogeneic transplantation of PDLSCs, the regeneration of the periodontium and healing of periodontitis were obtained based on the immune suppressive effects and their low immunogenicity [37]. A clinical study showed that autologous PDLSCs transplantation has long-term stability and efficacy advantages for patients with periodontitis, highlighting the increased potential of PDLSCs in the treatment of periodontitis [73].

### 3.2. Non-Dental Stem Cells

Non-dental stem cells are classified into BMSC, adipose-derived stem cells (ASCs), and embryonic stem cells (ESCs).

#### 3.2.1. Bone Marrow-Derived Mesenchymal Stem/Stromal Cells (BMSCs)

The recognition of the properties of marrow stromal cells has changed the perception of them, especially from a therapeutic perspective. BMSCs can differentiate into chondrocytes, osteoblasts, adipocytes, and muscle cells. BMSCs are classified according to the surface marker types (CD 29, CD 44, CD73, CD90, CD105, CD146, and STRO-1) and do not express CD14, CD34, or CD45. BMSCs have the ability to generate alveolar bone, Sharpey’s fibers, and cementum; hence, they can regenerate periodontal defects [74]. BMSCs can stimulate the expression of odontogenic genes and differentiate into osteoblasts and fibroblasts after systemic or local transplantation [75].

Another important characteristic of BMSCs is represented by their role in anti-inflammatory and immunosuppressive functions [47]. BMSCs mediate T cell proliferation by regulating immunomodulation. BMSCs inhibit inflammatory markers such as IL-1 and TNF-α, indicating that their use in the treatment of periodontitis could be feasible. Although remarkable improvements in periodontal parameters have been observed, clinical studies are needed to determine the role of BMSCs and their capacity to modulate inflammation and immunity before they become an option in the regenerative medicine/therapy of periodontitis [46].

#### 3.2.2. Adipose-Derived Stem Cells (ASCs)

ASCs are cells derived from adipose tissues and express markers similar to BMSCs, such as CD29, CD44, CD73, CD90, CD105, and CD166. However, ASCs do not express some markers specific to hematopoietic cells, such as CD31, CD34, and CD45 [76]. ASCs can improve the cementum and periodontal vessel regeneration and differentiate into adipocytes, osteocytes, and myogenic and neurogenic cells [1]. In comparison with BMSCs, ASCs have superior efficiency due to the easy harvesting process and the few notable complications at the donor site level. More importantly, ASCs, together with cytokines TNF-α, IFN-γ, and IL-6, drive the expression of immunosuppressive factors IL-1RA and GBP4 [49]. Previous work has shown that ASCs represent a potential candidate for periodontal treatment and regeneration (91). ASCs secrete growth factors such as insulin-like growth factor binding protein-6, which enable the differentiation of ASCs in the periodontium. An animal model study in which allogeneic ASCs were transplanted to the affected periodontal tissue showed the proliferation of novel PDL fibers, cementum, and alveolar bone [49].

### 3.3. Induced Pluripotent Stem Cells (iPSCs)

The use of human iPSCs in regenerative therapy represents an extremely useful solution, because they can solve problems related to the immune–rejection reaction and the ethical problems that arise when using ESCs. Somatic cells have been reprogrammed into iPSCs using different transcriptional markers such as Oct4, Sox2, and Krüppel-like factor 4. iPSCs express special markers TRA160, TRA180, CD73, CD90, CD105, CD146, and CD106 [77]. They contribute to the regeneration of alveolar bone formations, cementum, and regeneration of inflammatory tissue in periodontitis. Stem cells derived from dental tissue (buccal mucosal fibroblasts, gingival, apical papilla, and dental pulp) have the advantage of generating iPSCs [78].

iPSCs represent a potential source of cells used for prevention and treatments in regenerative medicine. A recent study showed that the transplantation of iPSCs onto an enamel-derived construct significantly increased the formation of alveolar bone, PDL, and cementum in an animal model with periodontal disease compared with groups where no iPSCs were used [71].

Various stem cell transplantation techniques have been used, such as direct injection of stem cells grown in culture medium at the site of the periodontal defect [36], using a sponge impregnated with stem cells, or injecting the culture medium with enriched stem cells [79]. The use of carriers to implant stem cells in the place of a periodontal defect reduces the viability and proliferation of these cells; therefore, the direct injection method is preferred [80].

## 4. Interaction between Stem Cells and the Periodontal Inflammatory Environment

Understanding the interaction mechanisms between stem cells and inflammation in periodontal disease is crucial for their subsequent use in periodontal regeneration [81]. For example, interactions between stem cells and immune cells are different in inflamed tissue during the regeneration process compared with healthy tissue [82]. Thus, it is important to recognize that stem cells have important immunomodulatory properties in inflamed periodontal tissues and that these properties are derived from inflamed tissue.

### 4.1. Stem Cells in the Inflammatory Environment

Stem cells derived from inflamed tissue show characteristics similar to stem cells, including colony formation, an increased proliferation rate, multilineage differentiation potential, and lower immunogenicity and immunosuppression [82].

Among stem cells, DMSCs derived from inflamed tissue possess certain advantages, namely, that they are more easily accessible and, from an ethical point of view, there are fewer complications [56]. Although PDLSCs are currently considered an ideal cell source for periodontal tissue regeneration compared with other DMSCs, obtaining these cells from healthy donors poses some challenges [83]. PDLSCs derived from inflamed periodontal tissues are considered iPDLSCs. Compared with PDLSCs, iPDLSCs have higher migratory and proliferative capacities. However, iPDLSCs are responsible for modifying the signaling pathways related to osteogenesis; for this reason, they show a lower osteogenic differentiation capacity [84]. These cells also have a decreased immunosuppressive potential and a reduced inhibitory capacity on T cell reproduction, PBMC proliferation, and Th17 differentiation compared with healthy tissue cells [85]. iPDLSCs secrete more TNF-α, IFN-γ, IL-2, and IDO, and less IL-10 [86]. When collagen sponges were combined with iPDLSCs isolated from inflamed human periodontal tissue in immunodeficient rats, this resulted in the formation of new collagen fibers, bone, and PDL-like tissue. Although the regeneration was not complete, important repair of the periodontal defects was still observed [72]. Compared with normal DPSCs, infected tissue-derived DPSCs (iDPSCs) show similar surface marker expression, proliferative properties, and multilineage differentiation potential [87]. The use of DPSCs derived from infected human tissue as a graft at the level of root furcation revealed novel alveolar bone formation [86]. These results may have important applications in periodontal regeneration with DMSCs obtained from inflammatory tissues [88]. In order to obtain optimal results in terms of periodontal regeneration, the implantation of DMSCs obtained from infected tissue should be carried out, avoiding the onset of periodontal pathological processes on healthy tissues as much as possible [89].

Nevertheless, future studies are needed prior to applying this procedure as standard protocol in a clinical setting. For example, the quality and quantity of stem cells depend on the source of the inflamed stem cells, the inflammatory state, and the experimental design [90]. In addition, inclusion and exclusion criteria, as well as the procedure for the isolation and grafting of inflamed stem cells, must be established and standardized. This way, the effects of inflamed stem cells in the periodontium can be monitored and evaluated in the long term by means of in vivo and in vitro experiments [29,89,91].

### 4.2. Infected Microenvironment and Its Influence on Stem Cells

The result of periodontal regeneration also depends on the interaction of stem cells with the adjacent infected environment, and the immunomodulatory activity of these cells is determined by the release of inflammatory cytokines into the circulation. Therefore, understanding the effects that inflammatory cytokines exert on stem cells is a very important aspect of optimizing and implementing clinical approaches mediated by stem cells [6,92]. Among the most effective inflammatory cytokines during the periodontal inflammatory process are TNF-α, IL-1β, IL-6, and IFN-γ [82], and they exert their effects by attenuating the immunosuppressive properties of stem cells [93]. Low levels of IFN-γ enhance the antigen presentation function of stem cells and thus reduce their lysis, whereas high levels have the opposite effect [94]. For example, an infected microenvironment produced by Porfiromonas gingivalis LPS led to significant improvement in the cell proliferation of DMSCs [95]. In addition, the co-culture of PDLSCs with IL-1β/TNF-α increased the proliferation rate of PDLSCs [84]. This differentiation potential of DMSCs has been shown to be mediated by pro-inflammatory cytokines and microbial pathogens [78]. Specifically, Porfiromonas gingivalis LPS and Escherichia coli LPS inhibit the osteoblastic differentiation of PDLSCs [96], whereas IL-1β/TNF-α levels in the microenvironment can determine the inhibition of the osteogenesis process [84].

In addition, there are other stem cells that play an important role through exerting important effects at the level of the infected microenvironment, such as BMSCs that require IFN-γ to cause immunosuppression on T lymphocyte proliferation. The transplantation of BMSCs in a culture medium stimulated by LPS inhibited the production of inflammatory cytokines, and reduced the destruction of the inflammatory tissue and obtained tissue regeneration [97]. Recent data have shown the regenerative potential of stem cells when used in an inflammatory environment [98]. This is due to their capacity for proliferation, migration, multiple cell differentiation and immunosuppressive and anti-inflammatory properties in the inflamed microenvironment [99]. Although still debatable, it seems that the use of stem cells in periodontal therapy provides best results after controlling the inflammation through non-surgical and surgical treatments [98].

## 5. Conclusions

Several studies conducted in recent years have shown that the use of stem cells in periodontal therapy has a promising potential due to the particular properties and molecular abilities of some of these cell types. Additionally, stem cells have remarkable versatility due to their state, proliferation, migration, multiline differentiation, and immunosuppressive characteristics. All of this, especially the immunomodulatory properties, make them attractive for use in the regenerative therapy of periodontal lesions. However, the therapeutic success depends on several factors that include the microenvironment, the source of stem cells, the experimental design, and the isolation and transplantation procedures of stem cells. In addition, the inclusion and exclusion criteria should be clearly defined, and the protocol for the isolation and transplantation of stem cells should be standardized for therapeutical application in periodontal tissue regeneration. Finally, clinical and preclinical studies are needed to determine the immunomodulatory mechanisms of stem cells in an inflammatory microenvironment, in order to enable the use of these cells in periodontal regenerative engineering.

## Figures and Tables

**Table 1:** Stem cells and multipotent differentiation at the level of distinct tissues.

Stem Cells	Multipotent Differentiation	Mechanism of Action	Therapeutic Intervention	Animal Experiment	Tissue Types	Reference
Dental Mesenchymal Stem Cells						
DFSCs	Cementoblasts Osteoblasts Chondrocytes	Migration, proliferation and osteogenic differentiation of PDLSCs	Transplantation of cell sheets with Treated Dentin Matrix complexes (TDM)	Experimental nude mice	Periodontal ligament (PDL) Cementum	[1,3,6,30,31,32]
DPSCs	Odontoblasts Osteoblasts Chondrocytes Adipocytes Neurogenic cells	Mediate both innate and the specific immune responses	Transplantation of cell sheets without additional materials	Experimental periodontitis in miniature pigs	Dentin pulp Blood vessel Neuronal	[6,29,33,34,35]
SCAPs	Osteoblasts Odontoblasts Adipocytes	Inhibit T lymphocyte proliferation	Local Injection	Minipig Model of Periodontitis	Dentin pulp Blood vessel	[2,29,36,37]
GMSCs	Osteoblasts Adipocytes Neurogenic cells	Inhibit the activity of M1 macrophages, reduce DC activation and maturation	Transplantation of a combination of GMSCs and hydroxyapatite synthetic extracellular matrix (HA-sECM)	Experimental periodontal defects in miniature pigs	Cementum Alveolar bone	[7,29,38,39,40]
SHEDs	Osteoblasts Adipocytes Myogenics Neurogenic cells	Mediate activation, maturation, and differentiation of T lymphocytes	Transplantation of cell sheets with TDM	Study on Sprague Dawley rats	Dentin pulp Blood vessel Neuronal	[1,3,6,36,41]
PDLSCs	Osteoblasts Cementoblasts Adipocytes Chondrocytes Neurogenic cells	Reduce the proliferation of PBMCs and secretion of glycoprotein 1b and PGE2 originating from dendritic cells	Transplantation of a combination of cells and materials	Experimental periodontitis in miniature pigs and canines	PDL Cementum	[4,5,40,42,43]
***Non-dental stem cells***						
BMSCs	Osteoblasts Odontoblasts Chondrocytes Adipocytes Neurogenic cells	Stimulate expression of odontogenic genes Anti-inflammatory and immunosuppressive functions	Injection of cell solution without additional materials	Experimental periodontitis in rats	Cartilage Bone Neuronal Muscle	[5,44,45,46,47]
ASCs	Osteoblasts Chondrocytes Adipocytes Neurogenic cells	Secrete growth factors (insulin-like growth factor binding protein-6	Cells Transplantation combined with platelet-rich plasma (PRP)	Periodontitis canine model	Cartilage Bone Blood vessel Adipose	[5,6,48,49,50]
Induced pluripotent stem cells						
iPSCs	Somatic cells		Transplantation of combined cells with scaffolds such as enamel matrix-derived (EMD) gel	Mouse experimental periodontitis	Bone Cartilage Myocardium Blood vessel Adipose Neuronal	[1,5,6,51,52]

## Abbreviations

DMSCs	*Dental mesenchymal stem cells*
iPSCs	*Induced pluripotent stem cells*
iDMSCs	*DMSCs derived from infected tissue*
PDL	*Periodontal ligament*
TLRs	*Toll-like receptors*
RANKL	*Receptor activator of nuclear factor kappa-B ligand*
IL	*Interleukin*
CCL	*Chemokine ligand*
CCR	*Chemokine receptor*
TNF-*α*	*Tumor necrosis factor α*
PDLSCs	*Periodontal ligament stem cells*
DFSCs	*Dental follicle stem cells*
DPSCs	*Dental-pulp-derived stem cells*
SCAPs	*Stem cells from apical papilla*
SHED	*Stem cells from exfoliated deciduous teeth*
GMSCs	*Gingival mesenchymal stem cells*
DSSCs	*Dental-socket-derived stem cells*
ALP	*Alkaline phosphatase*
PGE2	*Prostaglandin E2*
PBMCs	*Peripheral blood mononuclear cells*
IFN	*Interferon*
TGF-*ββ*	*Transforming growth factor-β*
IDO-1	*Indoleamine 2,3-dioxygenase-1*
HGF	*Hepatocyte growth factor*
PD-1	*Programmed cell death protein 1*
PD-L1	*Programmed cell death protein ligand 1*
HLA-ABC	*Human leukocyte antigen*
NK cells	*Natural killer cells*
MAPK	*Mitogen-activated protein kinase*
Tregs	*Regulatory T cells*
DCs	*Dendritic cells*
BMSCs	*Bone marrow stromal stem cells*
ASCs	*Adipose-tissue-derived stem cells*
iPDLSCs	*Inflammatory periodontal ligament stem cells*
ROS	*Reactive oxygen species*
MMPs	*Matrix metalloproteinases*
Th	*T helper cells*
TIMPs	*Tissue inhibitors of metalloproteinases*
CD	*Surface cluster of differentiation*
HLA-ABC	*Human leukocyte antigen*
NOTCH 1	*Neurogenic locus notch homolog protein 1*
LPS	*Lipopolysaccharides*
sEVs	*cell-derived small extracellular vesicles*
OPG	*Osteoprotegerin*
RANK	*Receptor activator of NF-κB*
HA-sECM	*Hydroxyapatite -synthetic extracellular matrix*
TDM	*Treated dentin matrix*
PRP	*Platelet-rich plasma*
EDM gel	*Enamel matrix-derived gel*
